# All-optical nonlinear activation function based on stimulated Brillouin scattering

**DOI:** 10.1515/nanoph-2024-0513

**Published:** 2025-02-14

**Authors:** Grigorii Slinkov, Steven Becker, Dirk Englund, Birgit Stiller

**Affiliations:** Max-Planck-Institute for the Science of Light, Staudtstr. 2, 91058 Erlangen, Germany; Department of Physics, Friedrich-Alexander-Universität Erlangen-Nürnberg, Staudtstr. 7, 91058 Erlangen, Germany; Research Laboratory of Electronics, Massachusetts Institute of Technology, Cambridge, MA 02139, USA; Institute of Photonics, Leibniz University Hannover, Welfengarten 1A, 30167 Hannover, Germany

**Keywords:** optoacoustics, nonlinear optics, nonlinear activation function, photonic neuromorphic computing, Brillouin scattering, optical fiber; optical neural network

## Abstract

Optical neural networks have demonstrated their potential to overcome the computational bottleneck of modern digital electronics. However, their development towards high-performing computing alternatives is hindered by one of the optical neural networks’ key components: the activation function. Most of the reported activation functions rely on opto-electronic conversion, sacrificing the unique advantages of photonics, such as resource-efficient coherent and frequency-multiplexed information encoding. Here, we experimentally demonstrate a photonic nonlinear activation function based on stimulated Brillouin scattering. It is coherent and frequency selective and can be tuned all-optically to take LeakyReLU, Sigmoid, and Quadratic shape. Our design compensates for the insertion loss automatically by providing net gain as high as 20 dB, paving the way for deep optical neural networks.

## 1 Introduction

Artificial neural networks (ANNs) have emerged as powerful instruments for solving difficult tasks that range from speech recognition to image processing in medicine. Thanks to their self-learning abilities and nonlinearity [1], they can provide creative solutions derived from their training on large data sets. After years of rapid scaling of model complexity, machine learning inference and training are close to reaching a bottleneck formed by the limitations of conventional Boolean logic processing hardware, especially with regard to power consumption, latency, and data movement.

Overcoming this “von Neumann” bottleneck has motivated the search for new ANN computing architectures based on fundamentally different principles. Transferring the linear algebraic operation – vector-matrix multiplication – of ANNs to the optical domain, in particular, yields a potential for fundamental improvements in energy consumption and latency [2], [3], [4], [5], [6], [7], [8], [9], [10], [11]. Although these approaches have demonstrated the potential of optical neural networks (NNs), most of them achieve nonlinearity through opto-electro-optic conversion or digital post-processing [5], [6], [7], [8], [9], [10], [12], [13], [14]. As the opto-electronic conversion of the signal at each neuron limits the power efficiency, the computing speed and the scalability of the system [15], all-optical activation functions are demanded [15], [16], [17], [18].

We propose a list of desirable, but so far not demonstrated in one “package”, features which is aimed at maximizing the benefits of having an all-optical activation function. These features are: (i) programmable nonlinearity, (ii) low insertion loss, (iii) coherence, (iv) WDM capability, and (v) compatibility with on-chip designs. The aim of this list is to ensure that the activation function will not impose additional limitations on the optical neural network as well as add new capabilities, allowing to fully exploit the potential of an optical neural network. A programmable activation function (i) allows the optical NN to adapt better to a specific problem and can be used as an additional training parameter. It has been shown for digital NNs that this additional degree of freedom is beneficial for the NN’s performance [19], [20]. Insertion losses (ii) limit the depth of the neural network, reducing the number of layers that can be stacked before the signal will have to be amplified. A coherent activation function (iii) is not only beneficial for phase-based optical NN architectures, such as [7], [9], but can also allow to implement efficient training schemes [21]. The WDM compatibility (iv) is essential for applying resource-efficient frequency-basis information encoding, which is a unique feature of photonics. Due to the overall lack of a frequency-selective activation function, multi-frequency photonic machine learning architectures have been so far limited to vector-matrix multiplication [10], [22], [23].

**Figure 1:**
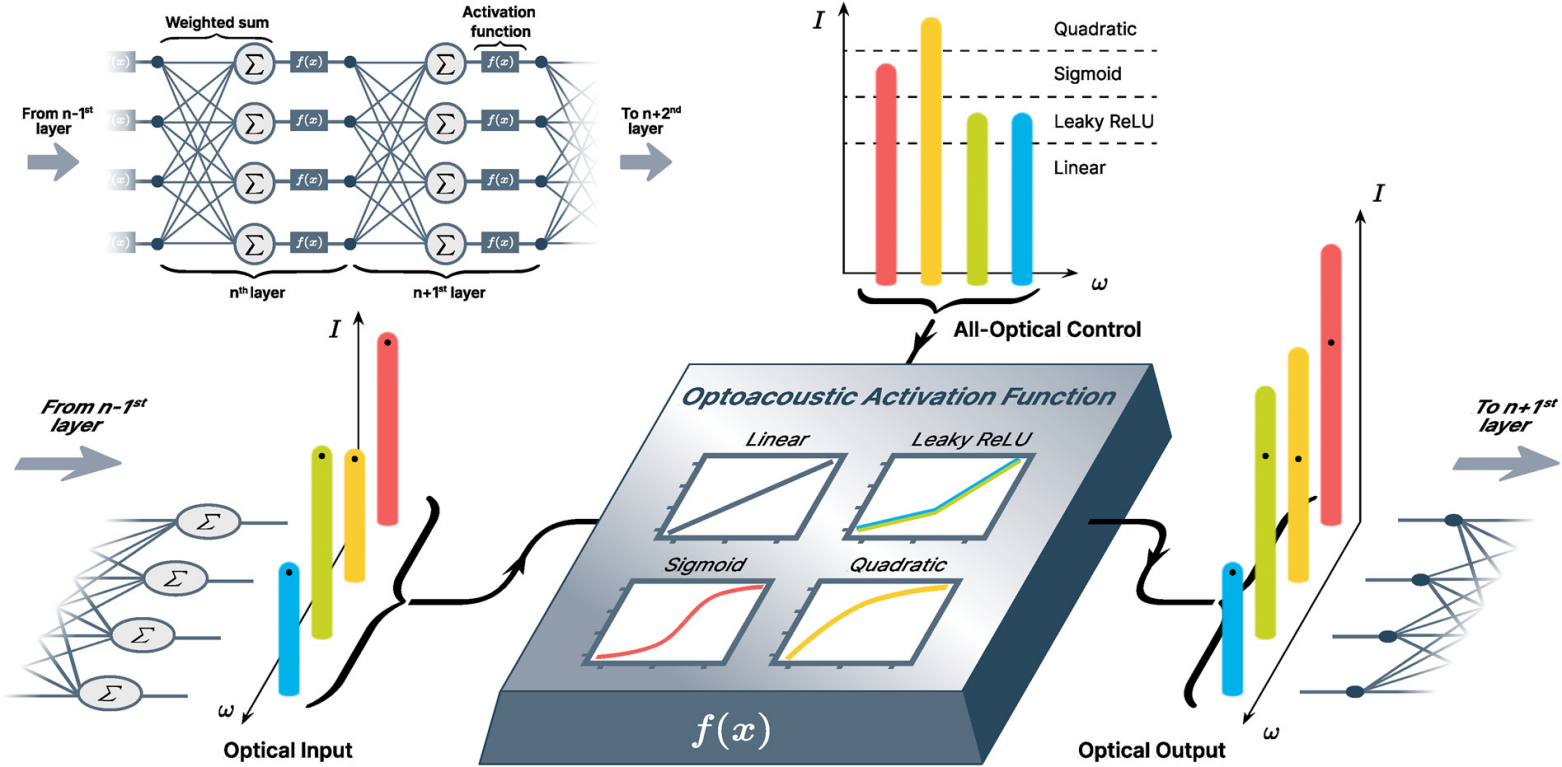
A schematic representation of how an optoacoustic activation function can be employed between layers *n* − 1 and *n* + 1 of an all-optical multi-frequency neural network. The result of the weighted summation of the *n* − 1 layer is encoded in the magnitudes of individual spectral components and sent to the optoacoustic activation function together with the multi-frequency control signal. The magnitudes of the control signal’s spectral components define the type of activation function applied to the corresponding input signal component. The optical output of the optoacoustic activation function has the same frequency components as the input, with their magnitudes transformed nonlinearly depending on the type of activation function. The output is fed to the next layer of the neural network. The inset shows a conceptual scheme of a neural network. Each neuron performs two crucial operations: it takes a weighted sum of the inputs and applies a nonlinear activation function to the result.

In this work, we experimentally demonstrate an optoacoustic activation function that combines features (i)–(v) (see Figure 1). Our design is based on the nonlinear effect of stimulated Brillouin scattering (SBS) [24], [25], [26], which arises from the interplay between optical and acoustic fields. SBS is inherently frequency-selective [27], which makes it particularly suitable for resource-efficient frequency-basis information encoding. This means that our all-optical activation function treats different frequencies independently, while being coherent. Moreover, our approach amplifies the signal with a positive net gain, facilitating its use in deep optical NNs. The nonlinear response is controlled all-optically and can be tuned continuously between different activation function shapes, including LeakyReLU, Sigmoid and Quadratic, which are favored by the machine learning community [5], [20].

While Brillouin scattering has been traditionally used for lasers [28], [29], [30], sensing [31], [32], gyroscopes [33], [34], and microscopy [35], [36], [37], with our work we reveal new applications, going beyond what has been previously demonstrated for Brillouin-based signal processing [38], [39], [40], [41], [42], [43], [44]. Our approach is not limited to a specific platform as SBS can be observed in different waveguide types ranging from optical chips [45], [46], [47], [48], [49], [50], [51], [52], [53] and microresonators [54], [55], [56], [57], [58] to photonic crystal fiber [39], [59], [60] and fiber tapers [61], [62].

In the following, we demonstrate different nonlinear input-output mappings of the photonic activation function and its tunability. In addition, we apply it to a dual-frequency signal, with a 3 GHz channel separation, demonstrating its frequency selectivity. Finally, we simulate the performance of our activation function using a digital neural network.

## 2 Results

### 2.1 SBS-based nonlinear activation function

**Figure 2:**
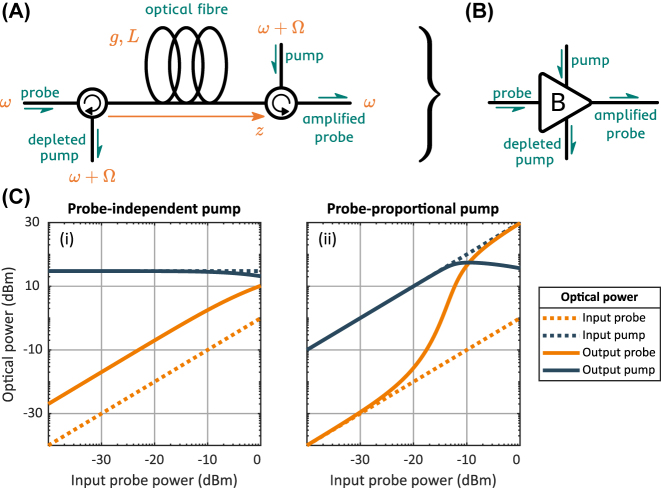
Brillouin amplifier schematics and modes of operation. (A) A schematic of an optical fiber-based Brillouin amplifier. (B) A convention introduced to represent the Brillouin amplifier. (C) Numerical simulation of SBS process, showing linear (i) and nonlinear (ii) relation between the input and the output probe optical power.

Stimulated Brillouin scattering (SBS) is a third-order nonlinear effect that couples a pair of counterpropagating optical waves with a traveling acoustic wave serving as a mediator between them [24]. It follows a strict phase-matching condition [27]: the frequencies of the optical waves, propagating in opposite directions, have to be separated by the acoustic wave’s frequency Ω, which, for a given optical wavelength, is defined by the properties of the interaction medium. A schematic of an experimental realization is depicted in Figure 2A, where the probe wave *a*_probe_ is taken to be the one with the lower frequency *ω* and the pump wave *a*_pump_ oscillates with the frequency *ω* + Ω. The interaction between the fields *a*_probe_, *a*_pump_, and *b* can be described formally with the interaction Hamiltonian (1) [63]:

(1) $${\hat{H}}_{\text{int}}=\hbar g\;\overset{\infty }{\underset{-\infty }{\int }}dz\;\left({\hat{a}}_{\text{pump}}\;{\hat{a}}_{\text{probe}}^{†}\;{\hat{b}}^{†}+\;H.c.\right),$$

with the optoacoustic coupling constant *g* and the time- and space-dependent wave packet operators ${\hat{a}}_{\text{probe}}\left(z,t\right)$ , ${\hat{a}}_{\text{pump}}\left(z,t\right)$ , $\hat{b}\left(z,t\right)$ of the probe, pump and acoustic field, respectively. This three-wave optoacoustic interaction holds at the quantum level [63], [64], [65] as well as in the classical regime [24], [26] and has been studied widely in optical fibers and on-chip configurations [39], [45], [46], [47], [48], [49], [50], [51], [52], [53], [54], [55], [56], [57], [58], [59], [60], [61], [62]. A detailed description of SBS can be found in the Supplementary material.

**Figure 3:**
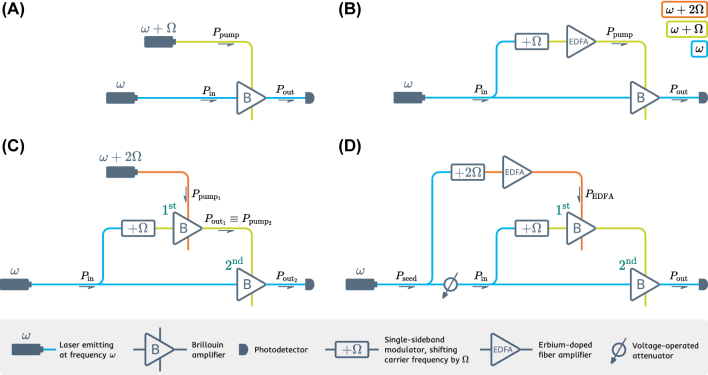
Schematic evolution of the activation function design. The steps that lead from the conventional Brillouin amplifier (A) and up to the double-stage scheme that is utilized in the experiment (D) are displayed.

Our nonlinear activation function is based on a modified Brillouin amplifier scheme (see Figure 3A) that has been demonstrated to deliver high-gain and low-noise operation [66], [67], [68]. Under the undepleted pump assumption (UPA), the equations governing SBS admit a simple analytic solution. UPA implies that the pump is not affected by the Brillouin interaction. In this case, the relation between the optical powers of the probe *P*_in_, the pump *P*_pump_ and the amplified probe *P*_out_ can be written as follows [25], [69]:

(2) $$P_{\text{out}}=P_{\text{in}}\;\exp\left(g\text{L}\cdot P_{\text{pump}}\right),$$

where *g* is the Brillouin gain and *L* is the interaction length.

We consider now a slightly different situation, where the usually independent pump power is made dependant on the optical input of the Brillouin amplifier: *P*_pump_ = *P*_pump_(*P*_in_) = *γP*_in_. Such a relation can be established using the scheme shown in Figure 3B, given that the chosen optical amplifier provides linear amplification. This way the information encoded in the intensity of the input light will be transferred to both the probe and the pump of the Brillouin amplifier.

Hence, the equation [2] would write as:

(3) $$P_{\text{out}}=P_{\text{in}}\;\exp\left(gL\cdot \gamma P_{\text{in}}\right).$$

Solving numerically the corresponding set of coupled mode equations for SBS allows us to study the behaviour of an input-dependent Brillouin amplifier for applying it as a versatile nonlinear activation function for an optical neural network architecture. The corresponding equations can be found in the Supplementary material.

The simulation results are presented in Figure 2C. The *g* ⋅ *L* product is taken to be 0.1 in our simulation. In both panels, the input probe power *P*_2_(0) is swept from −40 to 0 dBm. In the left panel, the input pump power *P*_pump_ is kept constant, which results in a linear dependence of probe output *P*_out_ on the probe input *P*_in_. As the output probe power surpasses −10 dBm, the pump starts to get depleted, and so the probe output growth rate decreases as well. In the right panel, both probe *P*_in_ and pump *P*_pump_ inputs are swept at the same rate. As one can see, this changes drastically the output probe dynamic: the dependence goes from linear to exponential growth and then back to linear, as we begin to observe the pump depletion. This nonlinear input-output relation in combination with the strict phase matching condition of SBS represents a perfect tool to implement a frequency-selective nonlinear activation function.

However, the scheme shown in Figure 3B had to be modified for the experiment. As can be seen in Figure 2C (ii), the nonlinear behavior of the output probe requires an input pump with high power and a high dynamic range. In order to satisfy this condition, we replace the EDFA with a Brillouin amplifier (see Figure 3C). In the created double-stage scheme, a Brillouin amplifier is pumped using the output of another Brillouin amplifier, both stages sharing the same probe. In order to increase the stability of the scheme, we utilize the same light source to create the pump for the first stage Brillouin amplifier, which comprises the final design (see Figure 3D). The information is encoded in the intensity of the input light using a voltage-operated attenuator placed after a portion of input has been utilized to create the pump.

### 2.2 Single-wavelength operation

**Figure 4:**
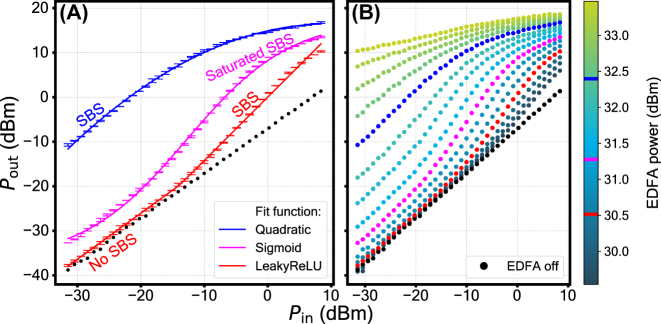
Nonlinear activation function shapes – the mapping between the optical input and the optical output of the setup. (A) A selection of curves fitted with conventional analytic activation functions. (B) The complete family of curves obtained at various 1st stage pump optical power levels provided by the EDFA.

The measurement results are presented in Figure 4, where the output power is plotted against the input power for different 1st stage pump power levels provided by an erbium-doped fiber amplifier (EDFA). Panel **A** shows a selection of activation function shapes accessible with the setup; panel **B** demonstrates the complete family of activation function curves. The variation of the EDFA power allows to choose a specific curve, adjusting the activation function shape continuously. When the EDFA is turned off, the activation function is linear.

The selected curves in panel **A** are fitted with analytical activation functions. A pump power of 30.5 dBm corresponds to the Leaky ReLU function. Its first section is a direct proportionality between the input and the output in the absence of SBS process – in this region the pump power is too low to be in the exponential regime of SBS. When the corresponding pump power exceeds the Brillouin threshold, the input light gets amplified. As a result, the input-output dynamic changes its slope as required for Leaky ReLU. The curve can be fitted with a polynomial of degree 4 (fit parameters are presented in section S8 of the supplement). The next curve, obtained at pump power of 31.3 dBm, is fitted with Sigmoid. Its nonmonotonic growth can be split into three distinct sections. First, the absence of SBS in the beginning. Second, the amplification provided by SBS in the middle section of the plot. Lastly, the saturated SBS in the final section, where the SBS process becomes so intense that the pump starts to get depleted, resulting in the saturation of the growth. The curve can be fitted with a polynomial of degree 3 (fit parameters are presented in section S8 of the supplement). The last curve in Figure 4A, obtained at 32.4 dBm pump power, is fitted with a Quadratic function, formed by a SBS process that gives way to saturated SBS as the probe power is increased. The curve can be fitted with a polynomial of degree 2 (fit parameters are presented in section S8 of the supplement). The optoacoustic activation function provides a gain of up to 2.4 dB, 8.8 dB, and 21.9 dB for the Leaky ReLU, Sigmoid, and Quadratic case, respectively. Hence, the optoacoustic activation function can be used to compensate for losses induced by the preceding matrix operation. This is an essential feature for implementing deep optical NNs.

**Figure 5:**
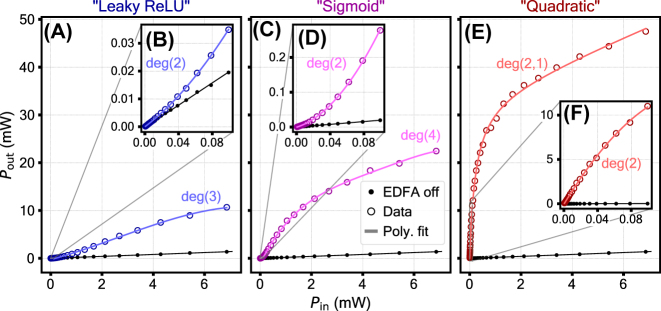
Nonlinear activation function shapes – the mapping between the optical input and the optical output of the setup. The polynomial fit degree *p* is specified as deg(p). Panels **A**, **C**, **E** show the full range of the input power used in the experiment. In panels **B**, **D**, **F** (insets) the lower-input parts of the activation functions are fitted separately. In panel **E** a rational function of order 2,1 (second degree polynomial divided by a first degree polynomial) is used. This is due to a nearly-asymptotic drop at low input power which cannot be fitted reasonably with a polynomial.

It is also useful to consider how the activation functions perform a linear scale. Figure 5 shows the same selection of curves as in Figure 4A: “Leaky ReLU” (30.5 dBm EDFA power), “Sigmoid” (31.3 dBm EDFA power) and “Quadratic” (32.4 dBm EDFA power). The experimental data is fitted with polynomial functions. The insets (panels **B**, **D**, **F**) are used to demonstrate the features found at low input power. The fit parameters can be found in the Supplementary material.

### 2.3 Dual-wavelength operation

**Figure 6:**
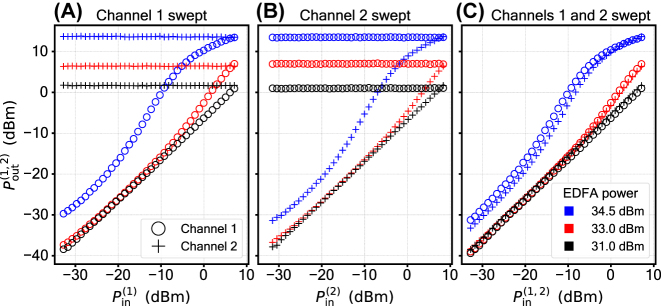
Demonstration of the frequency-selective operation. Circles and crosses correspond to a pair of wavelength-multiplexed channels, provided by two lasers at the input. The shape of the activation function in either of the channels is not affected by the SBS interaction taking place in the neighbouring channel. The instrumentation and the data acquisition process are identical between this measurement and the single-wavelength measurement, giving the same error level as in Figure 4.

We demonstrate the feature of frequency selectivity by splitting the input into two wavelength-multiplexed channels, provided by two tunable lasers at the input. The frequency separation between the two is set to 3 GHz, limited in the experiment by the spectrum analyzing device resolution. Each of the two channels hosts its own variable attenuator, which allows us to control the power levels independently (see supplement for details). We use a Finisar WaveAnalyzer to perform a wavelength-selective measurement of optical power at the output. The measurement results are plotted in Figure 6. Panel **A** shows the case where the power in channel 1 is swept, while the power in channel 2 is kept constant, panel **B** is vice versa. In panel **C**, both channels are swept simultaneously. The reference case where both channels are swept, but the EDFA is turned off can be found in the Supplementary material. The presented selection shows that for a given channel neither the presence, nor the variation of a signal in the neighbouring channel affects the shape of the nonlinear activation function. The EDFA power required to achieve the Sigmoid shape in the dual-wavelength case (34.5 dBm, Figure 6) is higher than what is needed to achieve the same shape in the single-wavelength case (Figure 4): 31.5 dBm. This is due to EDFA distributing its output power equally between the pumps of the two frequency channels, yielding a 3 dB less power per channel.

### 2.4 Simulated performance in a neural network

**Figure 7:**
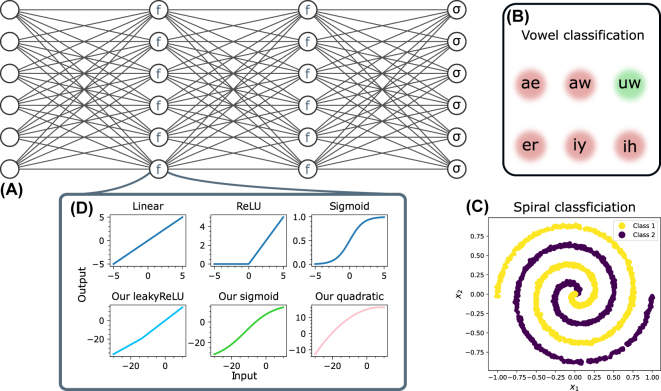
Testing our activation function in a digital neural network. (A) Three-layer neural network. We always use a softmax activation function in the output layer. Illustration of the neural network was created with [71]. (B) and (C) We apply the neural network for vowel and spiral classification, respectively. (D) We test the neural network with six different activation functions including the typical used ReLU and sigmoid functions.

In the following, we study how our activation functions could behave in an optical neural network. For this, we take references [12], [13], [14], [15], [70] as an example and equip a digital neural network with our activation function. We base our digital neural network architecture on experimentally demonstrated optical neural networks. More precisely, our digital network represents the size of one of the largest optical neural networks reported so far [9], which consists of three layers of six neurons each (see Figure 7A). In addition, we set the bias of all neurons to zero similar to the optical neural network. We evaluate the performance of the digital neural network using two tasks. Firstly, we perform a six class vowel recognition task as done in [9] (see Figure 7B). Secondly, we perform a binary classification task on a spiral dataset (see Figure 7C).

We perform each task with six different activation functions, one of which is a linear function (see Figure 7D). We take the digital implementations of the well-known ReLU and sigmoid functions and compare them to our LeakyReLU, Sigmoid, and quadratic realizations which are defined in the Supplementary material. We use a softmax activation function in the last layer of the digital neural network for all six cases.

To use our activation functions for the two tasks, we linearly transform the input data into the range [*p* − Δ/2, *p* + Δ/2], where *p* is the center position of the optical input and Δ is the dynamic range of the simulated optical input. With Δ, we assess how our activation functions perform for different input dynamic ranges. In the following studies, we use *p* = −13 and $\Delta \in \left\{6,\;10,\;20\right\}$ . More details about the underlying machine learning pipeline can be found in the Supplementary material.

#### 2.4.1 Vowel recognition task results

The vowel dataset is the one used in [72] and taken from [73]. Analogous to [9], we extract the formant frequencies *F*1, *F*2, and *F*3 at steady-state and at half of the vowel duration from the provided dataset. In total, the dataset provides 1,667 samples. After standardizing the values, we feed them into our pipeline.

**Figure 8:**
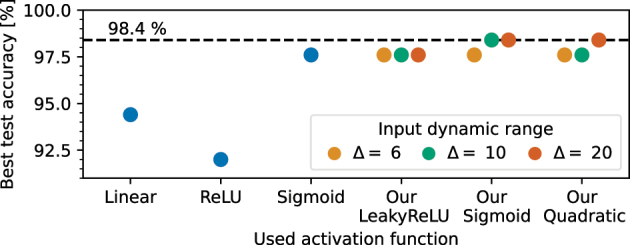
Best achieved test accuracy for the vowel task for the different activation functions.

Figure 8 shows the highest achieved test accuracy for the different activation functions selected from all swept parameters. Our activation functions perform as well as the digital sigmoid function. In fact, the neural networks based on our Sigmoid and Quadratic function achieved the highest training accuracy of 98.4 %. Furthermore, the results show that for this task the Δ value has only a little effect on the accuracy. Interestingly, the linear activation functions lead to a higher accuracy of 94.4 % than the ReLU activation function with 92.0 %.

One underlying reason for this could be the dying ReLU problem. This problem occurs when a neuron only outputs zero for any given input, which in turn leads to vanishing gradients [74]. In addition, it could be that the ReLU-based models are stuck in a local minimum for all hyper parameters configuration tested in this study. Consequently, this could lead to a lower accuracy as the one achieved by the models using a linear activation function.

As a result, the optimizer can no longer improve the neural network. Bandyopadhyay et al. achieved an accuracy of 92.7 % [9] which is slightly less than the results presented here. The reason for this could be that our digital neural network is not limited to unitary matrix operations. Details about the training dynamics can be found in the Supplementary material.

#### 2.4.2 Spiral classification task results

We generate the spiral data randomly starting by drawing an angle $\varphi_{0}\in \left[0,2\right)$ from a uniform distribution. This angle is then converted to the spiral coordinate (*x*, *y*) using

(4) $$\begin{array}{cccc} \varphi & =v\varphi_{0}, & r & =\frac{s\;\varphi_{0}}{2\pi },\\ x & =\left(r+\sigma_{\text{x}}\right)\;\cos\left(\varphi \right), & y & =\left(r+\sigma_{\text{y}}\right)\;\sin\left(\varphi \right), \end{array}$$

with the number of spiral turns *v*, the scale of the spiral *s*, and the noise on the *x* and *y* coordinates *σ*_x_ and *σ*_y_, respectively. For our study, we set *v* = 2, *s* = 1, and generate the noise *σ*_x, y_ with a Gaussian distribution with a mean value of 0 and with a standard deviation of 0.01. The resulting dataset is illustrated in Figure 7C. After sampling 1,000 data points, we follow the feature engineering approach of TensorFlow [75] and add the features *x* ^2^, *y* ^2^, and *x* ⋅ *y* to the dataset.

**Figure 9:**
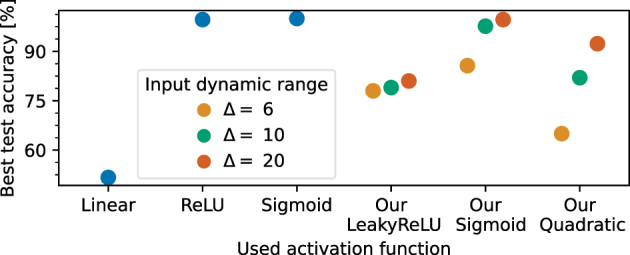
Best achieved test accuracy for the spiral task for the different activation functions.

Figure 9 shows the highest achieved accuracies for the different activation functions. The study shows that this task cannot be solved with high accuracy by a neural network equipped with the linear activation function. The best test accuracy achieved by such a model is 55.0 %, which is slightly above the random guessing accuracy of 50 %. In fact, the networks equipped with ReLU and Sigmoid activation functions achieved test accuracies of almost 100 %. Only our Sigmoid activation function achieves a similar accuracy. Moreover, our activation functions benefit from higher Δ values. In particular, models with our Quadratic function achieve significantly higher accuracies for higher Δ values. The reason for this could be that a higher degree of nonlinearity is provided for an input dynamic range. This could also explain why networks with our LeakyReLU function are less sensitive to different Δ values. A potential reason is the chosen center position of the optical input *p* which is close to the *P*_0_ parameter of our LeakyReLU function. Details about the training dynamics can be found in the Supplementary material.

## 3 Discussion

We have experimentally demonstrated for the first time, to the best of our knowledge, a nonlinear photonic activation function based on stimulated Brillouin scattering. Our activation function is coherent and frequency selective owing to the nature of SBS. A coherent activation function is the next step from the phase-reliant optical matrix multiplication approaches [7], [9] to fully all-optical ANNs that would not require opto-electronic conversion, which imposes bandwidth limitations, introduces cross-talk, requires introduction of time delay and eliminates frequency selectivity.

The frequency selectivity opens up prospects of drastically increasing the data throughput by exploiting wavelength multiplexing techniques to, for example, distribute the neurons in a layer across the frequency domain. It is the first demonstration of a frequency sensitive activation function, which shows no correlation between frequency channels that hinders existing solutions, such as frequency-encoded deep neural networks [10]. As another example, one can upgrade the throughout rate of the scheme presented by Shen et al. [7] using multi-frequency operation if an EDFA with sufficient output power is used. Naturally, the single input laser must be replaced with a multi-frequency input source such as multiple lasers or a frequency comb source. Then in theory each wavelength channel could be used to execute an independent training and inference step.

As it has been shown, there is no cross-talk between neighbouring frequency channels at frequency separations as small as 3 GHz, which surpasses the telecommunication standard of 25 GHz. For the continuous wave case, the minimal frequency separation between the two channels is intrinsically limited by the linewidth of the optoacoustic gain function, which, for the commercial single-mode fiber at room temperature is about 26 MHz [76]. As shown in [44], [76], the linewidth can be decreased by lowering the temperature of the waveguide. In the pulsed case, the minimal frequency separation is dictated by the pulse length [27]. This limitation goes both ways, meaning that the minimal pulse length is dictated by the channel separation. The general rule of thumb for SBS-based applications is this: channel separation has to be higher than Δ*ν*_laser_ + 1/*τ*_pulse_ + Δ*ν*_B_, where Δ*ν*_laser_ is the laser linewidth, *τ*_pulse_ is the pulse length and Δ*ν*_B_ is the acoustic gain linewidth.

The activation function can be tuned by varying the 1st Brillouin amplifier’s pump power to take such well-proven shapes as LeakyReLU, Sigmoid, and Quadratic.

With a numerical study, we have shown that our activation function shapes can achieve similar results in a digital neural network as their digital counterparts. In addition, the external control of the dynamics of the 1st stage Brillouin amplifier opens the possibility to use the nonlinearity as an additional training parameter of an optical ANN.

For digital ANNs, this has been shown to be a powerful tool for boosting the ANN performance [19], [20]. It is also feasible to engineer the 1st stage pump in such a way that different frequency channels would have different activation function shapes.

The output signal amplification that is inherent to our activation function design is suitable for compensating insertion and propagation losses. This should be particularly useful for designing deep optical NNs that comprise multiple neuron layers [21].

Though the experimental realisation presented in the paper relies on highly nonlinear optical fiber as the optoacoustic interaction medium, our approach is not limited to this platform. Conventional single-mode fiber (SMF) and photonic crystal core fiber (PCF) are also an eligible choice for SBS as well as integrated waveguides, including on-chip devices [41], [43], [77], [78], [79]. The choice of the platform combined with pulsed operation constitutes the way for improving the energy efficiency of the presented optoacoutic activation function. One needs to maximize the term *g* ⋅ *P*_pump_ *L*_eff_ − *αL* in order to improve the energy efficiency. Here, *P*_pump_ is the pump power, *L*_eff_ is the effective interaction length, and *α* is the optical loss of the waveguide (see Suppl. for details).

In conclusion, our frequency selective and coherent photonic nonlinear activation fills a gap in the current landscape of photonic machine learning. It could therefore be the key to unlocking the full potential of photonic neuromorphic computing.

## 4 Materials and methods

**Figure 10:**
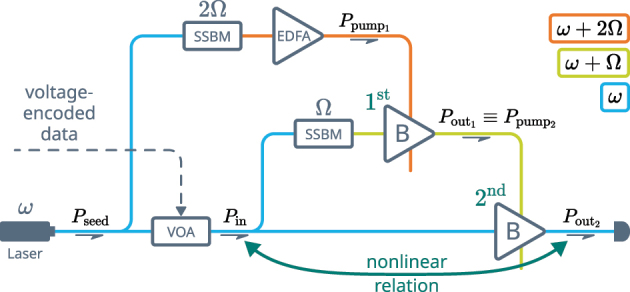
A principal scheme of the experimental setup: VOA – voltage-operated attenuator, SSBM – single-sideband modulator, EDFA – Erbium-doped fiber amplifier, B – Brillouin amplifier, as introduced in Figure 2B. The color of the connecting lines depicts the light frequency.

We implement the optoacoustic nonlinear activation using a setup depicted schematically in Figure 10. We build what can be called a double-stage Brillouin amplifier: the output of one Brillouin amplifier (1st stage) is utilized as a pump for another (2nd stage). The 1st stage Brillouin amplifier is pumped with an Erbium-doped fiber amplifier (EDFA).

The reason for pumping the 2nd stage with a Brillouin amplifier, as opposed to applying an EDFA directly, is that conventional EDFAs operate in the saturated regime, providing input-independent output power. This way, replacing the 1st stage with an EDFA would have eliminated any possible relation between the input and the pump that is required by [3]. The setup is fed with a 1550.12 nm fiber-coupled laser. A voltage-controlled attenuator (VOA) is used in the experiment to test the nonlinear input-output behaviour of the setup, simulating the amplitude-encoded data from the previous neuron layer. The VOA is inserted before the light gets distributed between the two stages, which ensures that they receive the same amplitude variation, as required by [3]. Note that feeding the top branch of the setup with the same laser is a measure taken to enhance the stability of the Brillouin amplifier and is not an actual requirement.

A Brillouin frequency shift *f*_B_ (corresponding angular frequency Ω = 2*πf*_B_) is applied to the middle branch of the setup, satisfying the SBS phase matching condition for the 2nd stage. This requires the signal in the top branch to be up-shifted by the sum of the Brillouin frequencies of the fibers. The optical fiber used in both of the stages was of the same material and structure, yielding a 2Ω shift for the top branch.

We use highly nonlinear fiber (HNLF) with equal parameters for the two Brillouin amplifiers. The lengths of the HNLF fibers used for the first and the second stage are 20 m and 100 m, correspondingly. The Brillouin frequency of the fibers is *f*_B_ = 9.730 GHz. A couple of single sideband modulators (SSBMs) driven with two separate RF sources apply required frequency shifts to the top and the middle branches of the setup.

## Supplementary Material

Supplementary Material Details
