# Supplementary material for: All-optical nonlinear activation function based on stimulated Brillouin scattering
Source: Nanophotonics. 2025 Feb 14;14(16):2711–22. doi: 10.1515/nanoph-2024-0513 (PMC12338876; doi:10.1515/nanoph-2024-0513)
Supplement: Supplementary file 1 — Supplementary Material Details [file j_nanoph-2024-0513_suppl_001.pdf]

Supplementary Information for All-optical nonlinear activation function based on stimulated Brillouin scattering

Grigorii Slinkov[1,*], Steven Becker[1,2,*], Dirk Englund[3], and Birgit Stiller[1,2,†]

[1]Max-Planck-Institute for the Science of Light,
Staudtstr. 2, 91058 Erlangen, Germany
[2]Department of Physics, Friedrich-Alexander-Universität Erlangen-Nürnberg,
Staudtstr. 7, 91058 Erlangen, Germany
[3]Research Laboratory of Electronics, Massachusetts Institute of Technology,
Cambridge, MA 02139, USA

[*]these authors contributed equally, [†]corresponding author: birgit.stiller@mpl.mpg.de

This PDF file includes:

Supplementary Text

Figs. S1 to S9

References 1 to 14

S1 Detailed description of Brillouin scattering

In the following, we describe the underlying dynamic of stimulated Brillouin scattering (SBS) using Fig. S1. A more detailed description can be found in References (*1–4*). The coincidence of the two counter-propagating optical fields a_{probe} at frequency ω_{probe} and a_{pump} at frequency

$\omega_{\text{pump}} = \omega_{\text{probe}} + \Omega$ in the waveguide creates an interference pattern. Through the effect of electrostriction, the interference pattern excites a traveling acoustic wave b. As the density variation is connected to the optical refractive index n of the medium, the optical waves are thus exposed to a moving Bragg-grating-like refractive index pattern. This causes an inelastic scattering of the a_{pump} into the probe wave a_{probe} and the acoustic wave b which then enhances the interference, substantiating the stimulated nature of the process.

S2 Extended illustration of the experimental setup

The extended experimental setup used for both the single and dual-frequency study is shown in Fig. S2. The setup is fed with a pair of lasers to simulate the frequency-multiplexed input. The separation between the two frequency channels $\omega_1 - \omega_2 \approx 3\,\text{GHz}$ is set by tuning the individual wavelengths of the lasers. For the single-wavelength study the setup was the same, but only one of the two lasers was used.

Each laser's output is split using a 50:50 coupler. One half is then used for the nonlinear activation function, whereas the other serves as a seed for the optical pump branch. The input power of the nonlinear activation function is controlled by a voltage-operated attenuator (VOA). In the dual-frequency experiment a second VOA is used for each of the two lasers, ensuring independent control of optical power for each of the frequency channels. The two frequency channels are combined using a 50:50 coupler. Their combination forms the activation function input, which will undergo the desired nonlinear transformation. The coupler acts also as splitter, feeding its input to the bottom and the middle branches of the setup which constitute the nonlinear activation function.

The (dual-frequency) signal in the lower branch serves as the input for the 2nd stage Brillouin amplifier based on a $100\,\text{m}$ long highly nonlinear fiber (HNLF).

The (dual-frequency) signal in the middle branch is first up-shifted by the Brillouin fre-

quency of the HNLF $f_\mathrm{B} = 9.730\,\mathrm{GHz}$ (corresponding angular frequency $\Omega = 2\pi f_\mathrm{B}$) with an optical IQ-modulator, which is configured to operate as a single-sideband modulator (SSBM). The resulting frequency up-shifted signal gets amplified by the 1st stage Brillouin amplifier based on a $20\,\mathrm{m}$ long HNLF. After that, it serves as the pump for the 2nd stage Brillouin amplifier.

The top branch of the setup provides the optical pump for the 1st stage Brillouin amplifier. Thus, it is seeded with the original laser combination, up-shifted by twice the Brillouin frequency and amplified by two cascaded Erbium-doped fiber amplifiers (EDFA). The first one acts as a pre-amplifier to seed the second high-power EDFA. Between the two EDFAs we apply a fixed value attenuator to comply with the input power limitations of the high-power EDFA. It is followed by a $1\,\mathrm{nm}$ bandpass filter to reduce the amplified spontaneous noise (ASE) level. A circulator isolates the EDFA from possible light backreflection and could be replaced with an optical isolator. The output power of the top branch is controlled with a third VOA for precise and quick tuning.

The output of the second Brillouin amplifier (2nd stage) forms the output of the nonlinear activation function. It is detected with a WaveAnalyzer (*5*) that performs a frequency-selective measurement.

S3 Dual channel input-output reference

Fig. S3 shows linear input-output dynamic for both frequency-multiplexed channels when the EDFA is turned off. It provides a reference for the Fig. 5 of the manuscript. Power in both channels is swept.

S4 Numerical study details

In a unidimensional waveguide (optical fibre) the equations governing SBS process can be written in terms of pump and probe optical power. Although these equations allow for an analytical solution (*6*), we follow the numerical approach discussed in (*2*). We solve the set of equations (1) in the conservative limit (no absorption):

$$\begin{cases} \frac{\partial P_1}{\partial z} &= -gP_1P_2 \\ \frac{\partial P_2}{\partial z} &= -gP_1P_2, \end{cases} \tag{1}$$

where $P_1 = P_1(z)$ and $P_2 = P_2(z)$ are the pump and the probe power measured along the fiber length z (Fig. S4), correspondingly, which means that following the conventions introduced in Section I of the main text,

$$\begin{cases} P_1(L) = P_{\text{pump}} \\ P_2(0) = P_{\text{in}} \end{cases} \tag{2}$$

Fig. 2 **C** of the main text presents the results of solving (1) with boundary conditions (2).

S5 Reducing the required power

In order to reduce the CW power required to pump the first Brillouin amplifier and therefore enhance the overall energy efficiency of the activation function, one can choose a longer waveguide and/or a waveguide with higher optoacoustic gain g. This will have two effects on the activation function.

Firstly, this will reduce the minimum pump power required to transition from the spontaneous to the stimulated regime. For a conventional Brillouin amplifier (such as the 1st stage in our scheme) this value corresponds to the "turn on" of the amplifier. For the 2nd stage amplifier in our scheme this would correspond to the transition point between the linear regime and the exponential growth ("no SBS" - "SBS") observed in Fig. 4 of the main text. This power can be expressed approximately as Equation (3) (*4*).

$$P_{\mathrm{th}} = \frac{21K}{gL_{\mathrm{eff}}}, \tag{3}$$

with the effective interaction length $L_{\mathrm{eff}} = (1 - \exp(-\alpha L))/\alpha$, the optical loss α, the effect of copolarized light $K = 3/2$ (*7*), and the peak Brillouin gain g. In our case, we can assume $\alpha_{\mathrm{HNLF}} = 0.28\,\mathrm{km}^{-1}$, $g_{\mathrm{HNLF}} = 1.25\,\mathrm{W}^{-1}\,\mathrm{m}^{-1}$ (*8*), which yields $P_{\mathrm{th}} = 1.26\,\mathrm{W}$ and $P_{\mathrm{th}} = 256\,\mathrm{mW}$ for the first and the second Brillouin amplifier, respectively.

Secondly, a higher gain-length product will reduce the amount of pump power required to achieve gain values similar to those demonstrated. In the simplest case, the gain of a Brillouin amplifier can be written as in Equation (S.4).

$$g_{\mathrm{amp}} = \exp(gL_{\mathrm{eff}}P_{\mathrm{pump}} - \alpha L) \tag{4}$$

with the intrinsic Brillouin gain g and the waveguide length L. Accordingly, the pump power P_{pump} can be reduced by increasing the Brillouin gain g and L_{eff}. This can be achieved by using a chalcogenide fiber or an integrated waveguide, such as a rib chip or, alternatively, a photonic crystal fiber (*9*, *10*).

The current HNLF-based design with the first Brillouin amplifier length $L = 20\,\mathrm{m}$ offers $10\log_{10}(g_{\mathrm{amp}}) \approx 54\,\mathrm{dB}$ gain when driven with $P_{\mathrm{pump}} = 500\,\mathrm{mW}$.

In a chalcogenide waveguide, given the experimentally demonstrated parameters $g_{\mathrm{AsS}} = 500\,\mathrm{W}^{-1}\,\mathrm{m}^{-1}$, $\alpha_{\mathrm{AsS}} = 1.2\,\mathrm{km}^{-1}$ and chip length of $L_{\mathrm{AsS}} = 0.2\,\mathrm{m}$ (*9*, *11*), the same gain can be achieved with 24.5 % less pump power required: $P_{\mathrm{pump,AsS}} \approx 0.38\,\mathrm{W}$. Note that chip designs with g_{AsS} as high as $750\,\mathrm{W}^{-1}\,\mathrm{m}^{-1}$ have been shown experimentally, however with a shorter waveguide length (*12*).

S6 Latency of the activation function

The latency of the optoacoustic nonlinear activation function (the minimal delay between two consecutive pulses) τ_{NAF} is defined by the combination of the equilibrium time of the system τ_{eqi} and the time-of-flight τ_{ToF}.

The equilibrium time is required to ensure the perfect overlap between the pump and the probe (with regard to the 2nd stage) pulses, hence $\tau_{\mathrm{eqi}} = n_{\mathrm{eff}}/c_0 \cdot (L_{\mathrm{HNLF}_1} + L_{\mathrm{HNLF}_2} + L_{\mathrm{devices}_1})$, where c_0 is the speed of light, n_{eff} is the effective refractive index of the fiber, L_{HNLF_1} and L_{HNLF_2} are the lengths of the 1st and the 2nd stage fibers, L_{devices_1} is the accumulated length of the devices (such as the SSBM) and the connecting fibers the pump pulse has to traverse.

The time-of-flight is required by the optical input pulse to reach the end of the activation function. It can be formally defined as $\tau_{\mathrm{ToF}} = n_{\mathrm{eff}}/c_0 \cdot (L_{\mathrm{HNLF}_2} + L_{\mathrm{devices}_2})$, where L_{devices_2} is the accumulated length of the connecting fibers in the probe pulse path.

In total, the latency of the activation function with regard to the variation of the input is $\tau_{\mathrm{NAF}} = \tau_{\mathrm{eqi}} + \tau_{\mathrm{ToF}}$. We estimate the lengths of the connecting fibers and devices to be $L_{\mathrm{devices}_1} \approx 15\,\mathrm{m}$ and $L_{\mathrm{devices}_2} \approx 10\,\mathrm{m}$. This gives us total equilibrium time $\tau_{\mathrm{eqi}} \approx 648\,\mathrm{ns}$ and the time-of-flight $\tau_{\mathrm{ToF}} \approx 528\,\mathrm{ns}$, yielding overall latency $\tau_{\mathrm{NAF}} \approx 1.2\,\mu\mathrm{s}$.

S7 Fit results

In order to proof the similarity of the obtained activation function shapes we fit the measurement data with a Leaky ReLU, sigmoid, and quadratic function. Firstly, we fit the function defined in Equation (5) using the function OPTIMIZE.CURVE_FIT of the PYTHON package SCIPY (*13*). The corresponding fit parameters are shown in Equation (5). The fit errors are extracted by taking the square root of the diagonal elements of the covariance matrix. Note that we rounded

the error of the parameter P_0 up to match the significant digits.

$$\text{LeakyReLU}(P_{\text{in}}) = \begin{cases} m_1\,(P_{\text{in}} - P_0) + b, & P_{\text{in}} < P_0 \\ m_2\,(P_{\text{in}} - P_0) + b, & P_{\text{in}} \geq P_0 \end{cases}, \tag{5}$$

$$P_0 = -13.84 \pm 0.79, \quad b = -19.65 \pm 0.98, \quad m_1 = 1.03 \pm 0.02, \quad m_2 = 1.42 \pm 0.02.$$

The next curve, obtained at an EDFA power of $31.3\,\text{dBm}$, is fitted with SIGMOID $\sigma(P_{\text{in}})$ (see Equation (6)). The corresponding fit parameters are shown in Equation (6). Note that we rounded up the error of the parameter b to match the significant digits.

$$\sigma(P_{\text{in}}) = \frac{a}{1 + \exp(-b\,(P_{\text{in}} - P_0))} + c, \tag{6}$$

$$P_0 = -12.80 \pm 0.20, \quad a = 54.76 \pm 1.18, \quad b = 0.12 \pm 0.01, \quad c = -36.92 \pm 0.70.$$

The last curve in Fig. 4 **A** of the main text, obtained at an EDFA power of $32.4\,\text{dBm}$, is fitted with a QUADRATIC function, formed by the saturation of the Brillouin process for increased input powers. Equation (7) shows the fit results. Note that we rounded the error of the parameter a up to match the significant digits.

$$f(P_{\text{in}}) = aP_{\text{in}}^2 + bP_{\text{in}} + c, \tag{7}$$

$$a = -0.02 \pm 0.01, \quad b = 0.32 \pm 0.01, \quad c = 14.91 \pm 0.07.$$

S8 Polynomial Degree Study

In order to quantify the nonlinearty of our activation functions, we fit our data with polynomials $f(x) = a_n x^n + a_{n-1} x^{n-1} \cdots + a_1 x + a_0$ of degree n. The minimal required polynomial degree n

Table S1: Polynomial fit results for the data shown in Figure S5. The units of the fit parameters are dropped as the fit function does not represent a real physical entity.

Function	$a_4 \times 10^5$	$a_3 \times 10^3$	$a_2 \times 10^2$	a_1	a_0
Qudratic			-1.66 ± 0.04	0.32 ± 0.01	14.91 ± 0.07
Sigmoid		-1.14 ± 0.06	-4.40 ± 0.22	1.02 ± 0.02	8.41 ± 0.14
LeakyReLU	-3.73 ± 0.22	-2.01 ± 0.11	-1.83 ± 0.13	1.49 ± 0.01	0.64 ± 0.06

required to sufficiently represent our measured data, quantifies the nonlinearity of the activation function. We analyze the nonlinearty of our Leaky ReLU, Sigmoid, Quadratic function. Therefore, we fit the data with polynomials of different degrees and check whether the fit represents the data well. As long as the chosen degree is not sufficient, we increase it until we find the minimal degree that achieves a sufficient agreement with our data. The fits are done with the function NUMPY.POLYFIT.

Figure S5 shows the results for different activation functions. The corresponding fit parameters are listed in Table S1. The polynomial fit confirms that our Quadratic function represents a polynomial of degree 2. The Sigmoid function requires a polynomial of degree 3 to be fitted well. The highest degree requires our LeakyReLU function. With a polynomial of degree 4 it is possible to parameterize the rich nonlinearties of the curve.

S9 Fit Results in Linear Scale

In Figure 5 of the main text, we discuss the dynamics of our activation functions when representing the data in units of mW instead of dBm. Therefore, we fit the data either with polynomials, $f(x) = a_n x^n + a_{n-1} x^{n-1} \cdots + a_1 x + a_0$, or with a rational function, $f(x) = P(x)/Q(x) = (p_n x^n + p_{n-1} x^{n-1} \cdots + p_1 x + p_0) \, / \, (q_n x^n + q_{n-1} x^{n-1} \cdots + q_1 x + 1)$. Note that $Q(x) \neq 0 \; \forall x$ due to the definition $q_0 = 1$.

In addition, we perform the fits selectively for a low input power regime ($P_{\text{in}} < 1\,\mathrm{mW}$) and

Table S2: Polynomial fit results for the data shown in the insets of Figure 5 of the main text ($P_{\text{in}} < 1\,\text{mW}$). The units of the fit parameters are dropped as the fit function does not represent a real physical entity.

Function	a_2	a_1	a_0
"LeakyReLU"	$1.24 \times 10^{0} \pm 0$	$2.29 \times 10^{-1} \pm 0$	$9.65 \times 10^{-6} \pm 2.65 \times 10^{-5}$
"Sigmoid"	$2.07 \times 10^{1} \pm 0$	$7.79 \times 10^{-1} \pm 0$	$-5.11 \times 10^{-4} \pm 2.44 \times 10^{-4}$
"Qudratic"	$-4.71 \times 10^{2} \pm 0$	$2.57 \times 10^{2} \pm 0$	$-5.44 \times 10^{-2} \pm 1.63 \times 10^{-2}$

Table S3: Polynomial fit results for the data shown in Figure 5 of the main text ($P_{\text{in}} > 1\,\text{mW}$). The units of the fit parameters are dropped as the fit function does not represent a real physical entity.

Function	$a_4 \times 10^2$	$a_3 \times 10^1$	a_2	a_1	a_0
"LeakyReLU"		-0.25 ± 0.04	0.20 ± 0.04	1.42 ± 0.08	-0.28 ± 0.03
"Sigmoid"	-2.02 ± 0.40	3.80 ± 0.51	-2.70 ± 0.20	10.61 ± 0.20	-1.02 ± 0.05

a high power regime ($P_{\text{in}} \geq 1\,\text{mW}$). The reason for this is the rich dynamic of the measurement data.

Table S2 lists the fit parameters for the low input power regime ($P_in < 1\,\text{mW}$). Note that we set the errors of the parameters in Table S2 to zero if they are significantly lower than the value of the corresponding parameter itself. Table S3 lists the polynomial fit parameters for the high power regime ($P_in \geq 1\,\text{mW}$). The parameters of the rational function fit used for the "Quadratic" curve are shown in Table S4. Note that $q_0 = 1$ due to the definition of the rational function.

Table S4: Rational function fit results for the "Quadratic" data shown in Figure 5 of the main text ($P_{\text{in}} \geq 1\,\text{mW}$). The units of the fit parameters are dropped as the fit function does not represent a real physical entity.

	p_2 or q_2	p_1 or q_1	p_0 or q_0
$P(x)$	4.44 ± 0.97	2.54 ± 0.35	4.65 ± 1.17
$Q(x)$		96.3 ± 10.8	1

S10 Machine Learning Pipeline

We implement the digital neural network using TENSORFLOW (v.2.16.1) (*14*). We train the three-layer neural network for 2500 epochs for the vowel recognition task and for 5000 epochs for the spiral task. For both cases, we use an Adam optimizer and a cross-entropy loss. We employ $70\,\%$ of the corresponding dataset for training and $15\,\%$ for validation and testing respectively.

We evaluate each network for three different learning rates $lr \in \{0.001,\ 0.0005,\ 0.0001\}$. In order to ensure reproducibility, we set the random seed of the python packages TENSORFLOW, NUMPY, and RANDOM at the beginning of each run. In total, we train each network with ten different seeds: 1, 3, 6, 8, 13, 30, 88, 99, 101, 1996.

S11 Training dynamics for the vowel classification task

Fig. S6 shows the training dynamics of the different models. The error band narrowing for higher training epochs of our LeakyReLU function indicate that these models seem to converge faster. For example, the error band of the networks with the digital sigmoid function also narrows, however has not yet reached a steady state regime as the study of our LeakyReLU.

S12 Training dynamics for the spiral classification task

The training dynamics for all sweeps are shown in Fig. S7. Compared to the training dynamics of the vowel classification task shown in Fig. S6, we can observe that the error band is larger and does not decrease for higher training epochs. This shows the sensitivity of the task to the chosen hyperparameter. In particular, the network trained with our quadratic function shows two validation accuracy levels. As a consequence, an extended hyperparameter optimization could improve the performance of the networks equipped with our activation functions.

S13 Frequency batching for wavelength division multiplexing operation

As indicated in Fig. S8 an optical pump can in principle interact with two frequency channels. However, this fact leads to competing Brillouin interaction which prevents a channel independent operation of the optoacoustic activation function. Fig. S9 shows a configuration with such a competing Brillouin interaction for a wavelength division multiplexing operation with a channel seperation ω_{ch}. Here, the optical input at ω_0 would amplify and attenuate the target and it's twin channel, respectively, since both channels are separated to the pump by the Brillouin frequency Ω.

The introduction of frequency batches suppresses those unwanted twin interactions and ensures a frequency selective operation of the optoacoustic activation function (see Fig. S10). Each frequency batch is separated by a batching frequency $\omega_{\mathrm{batch}} \neq l \cdot \omega_{\mathrm{ch}}$, $l \in \mathbb{N}$. The minimal value for ω_{batch} is determined by the intrinsic Brillouin linewidth Γ_{B}: $\omega_{\mathrm{batch}} \gg \Gamma_{\mathrm{B}}$. Due to the missing twin channel (see Fig. S10), the optical weight can only interact with the target channel. Moreover, no frequency batching is required if one choses the channel seperation to be $\omega_{\mathrm{ch}} \notin [2\Omega/k - \Gamma_{\mathrm{B}},\, 2\Omega/k + \Gamma_{\mathrm{B}}]\,,\, \forall k \in \mathbb{N}$.

S14 References

1. C. Wolff, M. J. A. Smith, B. Stiller, C. G. Poulton, *Journal of the Optical Society of America B* **38**, 1243 (2021).
2. R. W. Boyd, *Nonlinear optics* (Academic Press, Amsterdam ; Boston, 3rd ed, 2008), 613 pp.
3. A. Kobyakov, M. Sauer, D. Chowdhury, *Advances in Optics and Photonics* **2**, 1 (2010).
4. G. Agrawal, in *Nonlinear Fiber Optics* (Elsevier, 2013), pp. 353–396.
5. *WaveAnalyzer 1500S High-Resolution Optical Spectrum Analyzer — Coherent Corp.* (2023; `https://ii-vi.com/product/waveanalyzer-1500s-high-resolution-optical-spectrum-analyzer/`).

6. F. S. Gökhan, H. Göktaş, V. J. Sorger, *Applied Optics* **57**, 607 (2018).
7. M. Van Deventer, A. Boot, *Journal of Lightwave Technology* **12**, 585–590 (1994).
8. S. Becker, A. Geilen, B. Stiller, *High-speed coherent photonic random-access memory in long-lasting sound waves*, 2023, arXiv: `2311.06219[physics]`.
9. B. J. Eggleton, C. G. Poulton, P. T. Rakich, M. J. Steel, G. Bahl, *Nature Photonics* **13**, 664–677 (2019).
10. L. B. Martínez, P. Wiedemann, C. Zhu, A. Geilen, B. Stiller, *Optoacoustic cooling of traveling hypersound waves*, 2023, arXiv: `2305.19823[physics,physics:quant-ph]`.
11. A. Choudhary *et al.*, *Journal of Lightwave Technology* **35**, 846–854 (2017).
12. B. Morrison *et al.*, *Optica* **4**, 847 (2017).
13. P. Virtanen *et al.*, *Nature Methods* **17**, 261–272 (2020).
14. M. Abadi *et al.*, *TensorFlow: Large-Scale Machine Learning on Heterogeneous Distributed Systems*, 2016, arXiv: `1603.04467`.

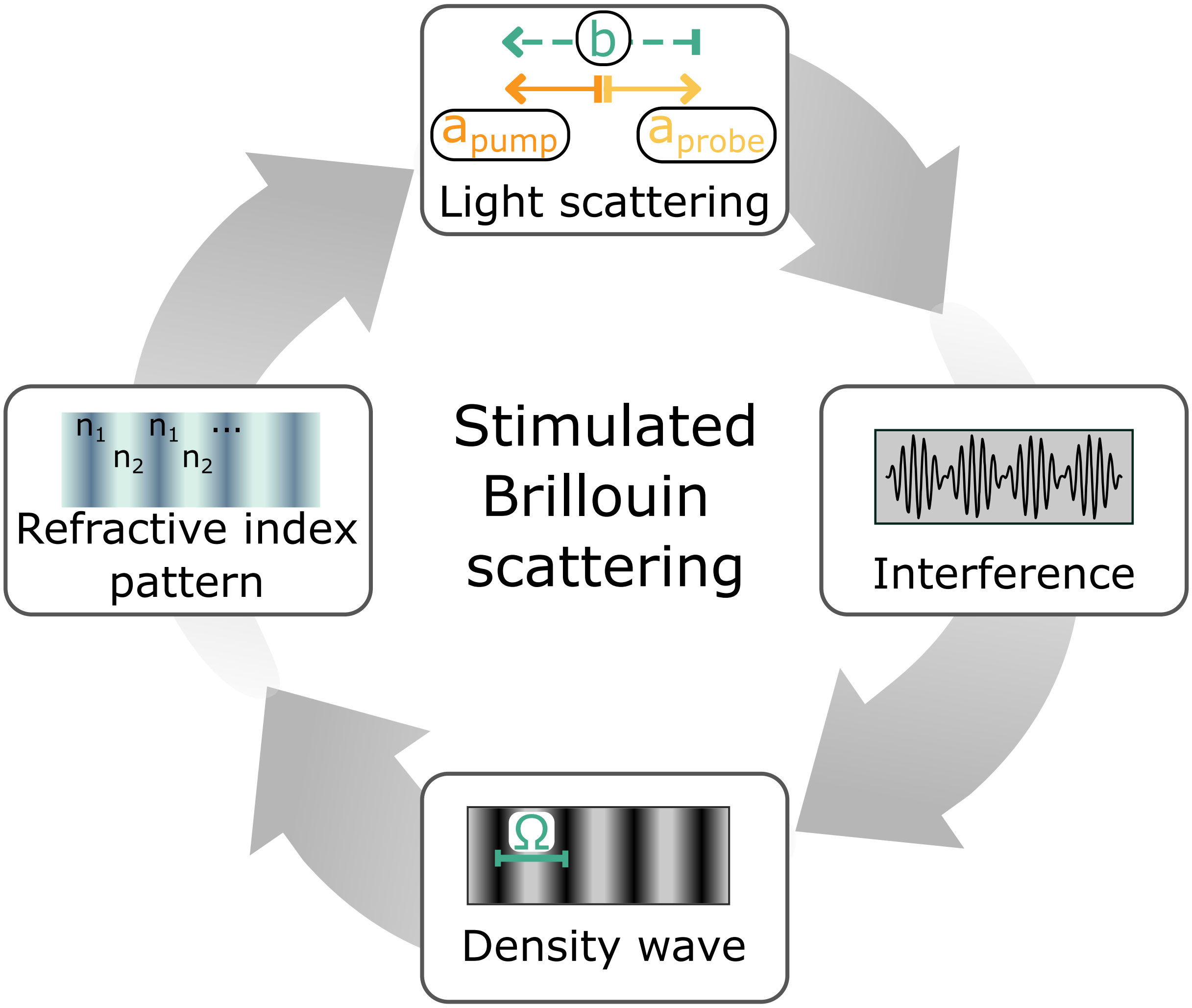

Fig. S1: Illustrated of the SBS feedback mechanism Initially, light waves ω_{probe} and a_{pump} scatter off acoustic phonons b, experiencing a frequency shift known as Brillouin frequency Ω. The interplay between the incident and backscattered light fields generates a dynamic interference pattern in motion. Electrosriction then translates this pattern into a mobile density wave, changing the refractive index n of the medium due to the photoelastic effect. This resulting density wave augments scattering efficiency, enabling a stimulated process.

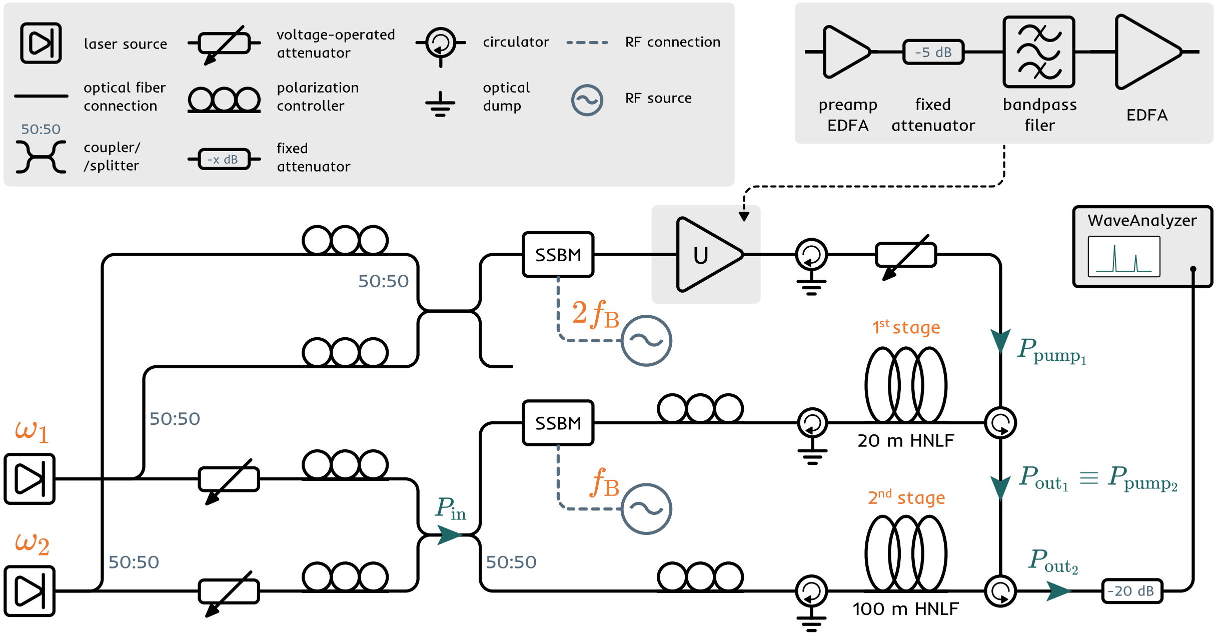

Fig. S2: Extended illustration of the experimental setup used in the study. In the single-frequency case one of the lasers was turned off.

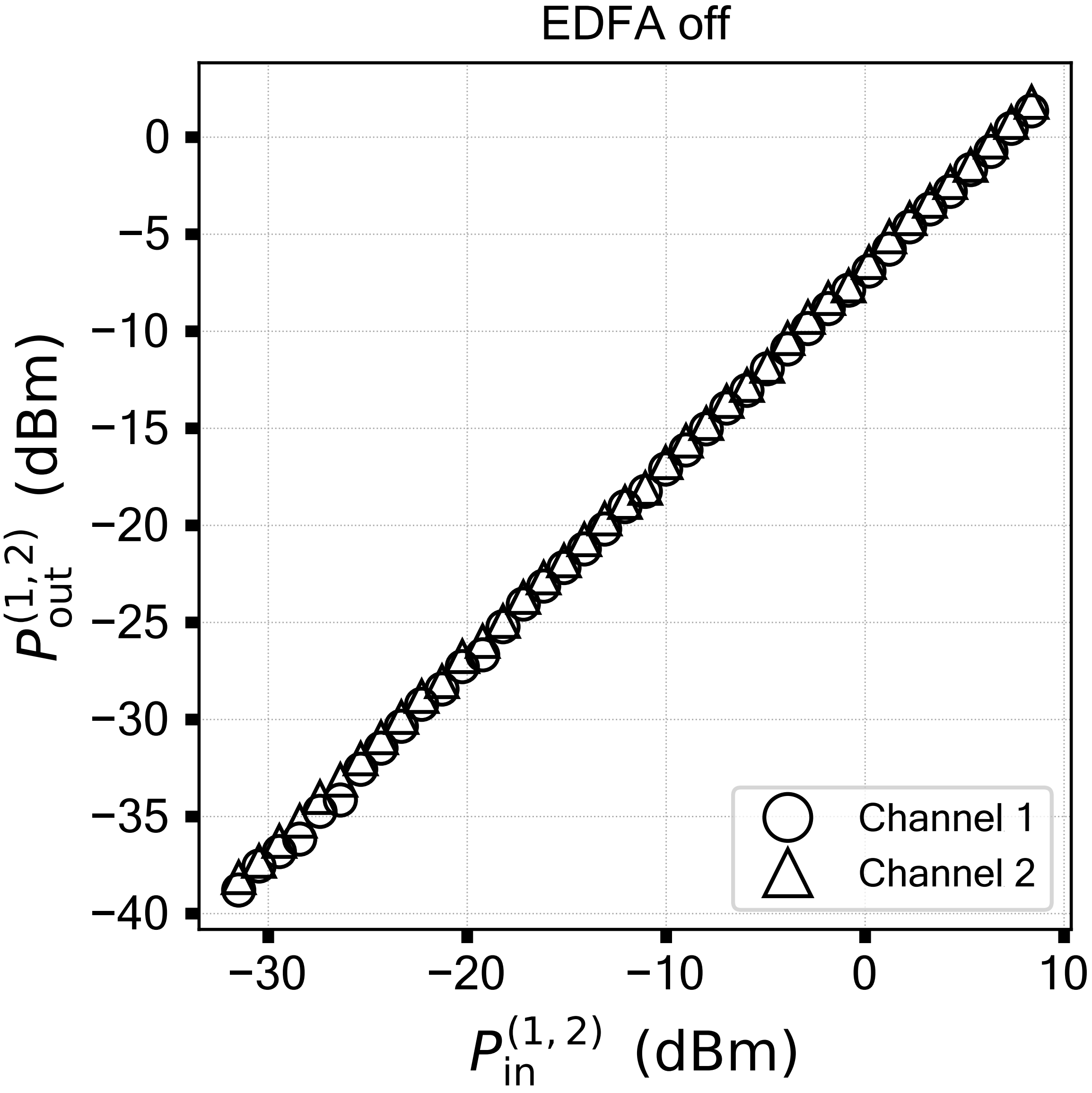

Fig. S3: Dual-frequency operation with the EDFA turned off.

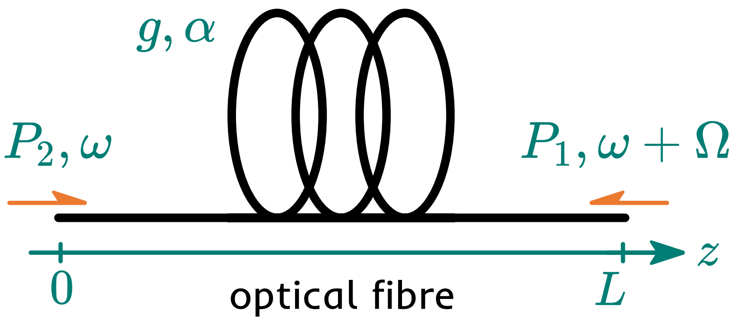

Fig. S4: Brillouin process in an optical fiber. P_2 is the probe wave, P_1 is the pump wave.

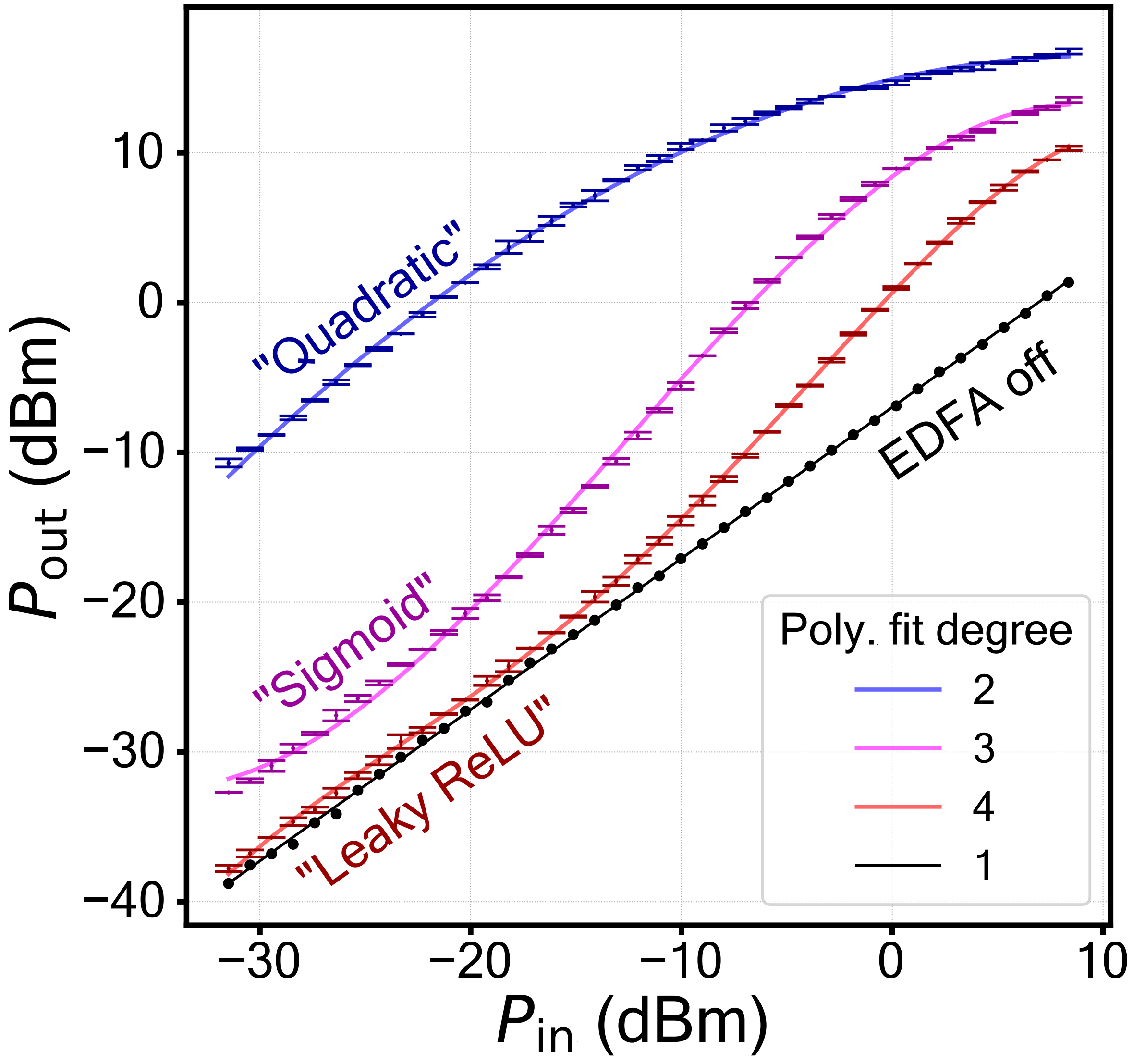

Fig. S5: Polynomial fit of the measured activation functions. The corresponding fit parameters are shown in Table S1.

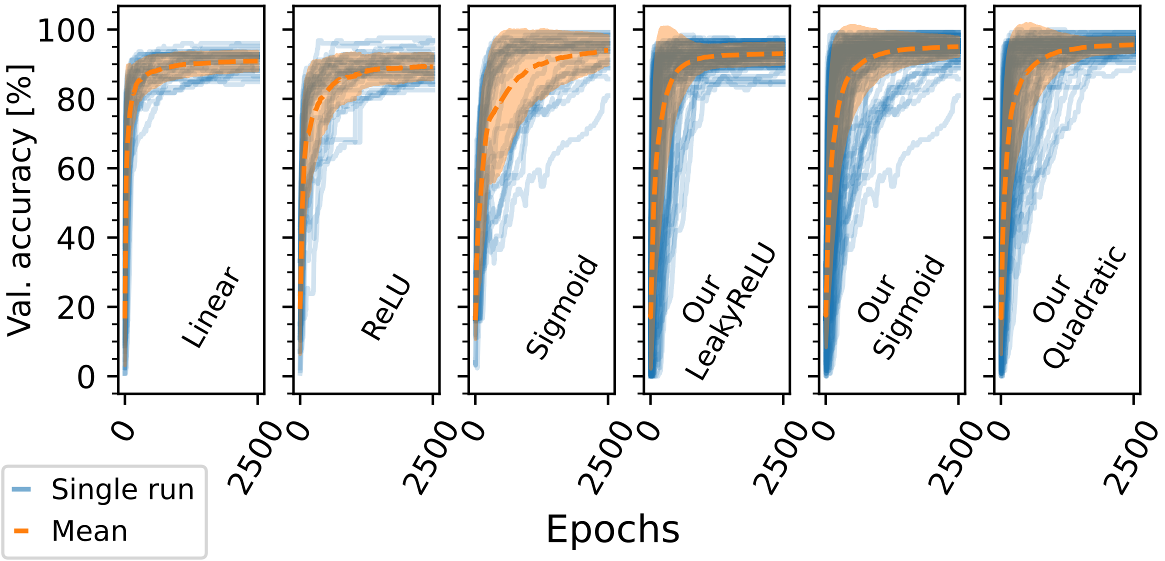

Fig. S6: Training dynamics of the vowel task. The dashed line shows the mean behavior. The corresponding standard deviation is illustrated as an error band.

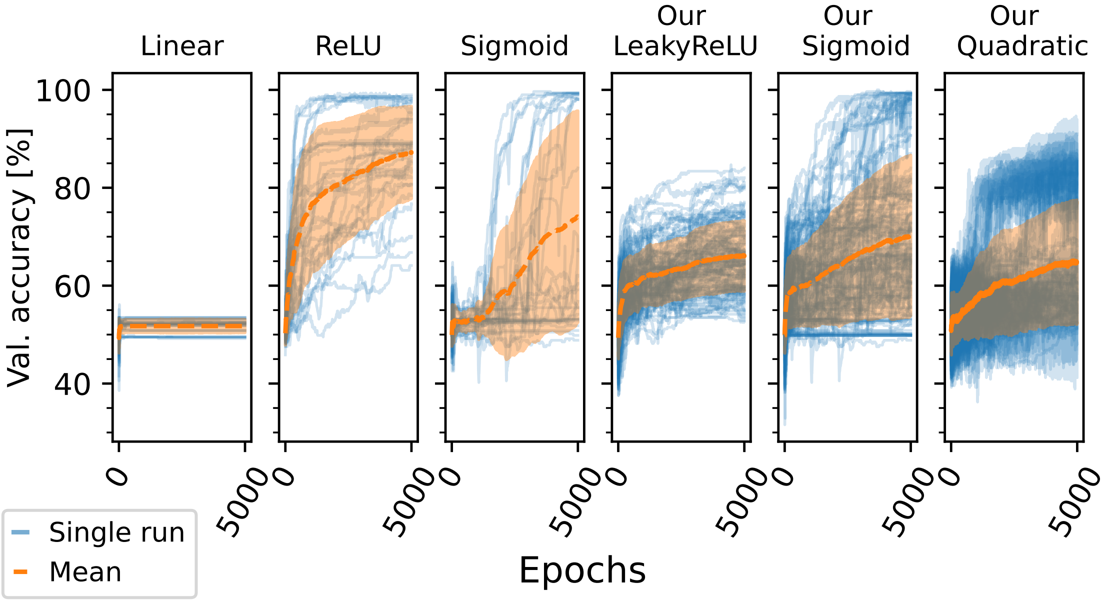

Fig. S7: Training dynamics of the spiral task. The dashed line shows the mean behavior. The corresponding standard deviation is illustrated as error band.

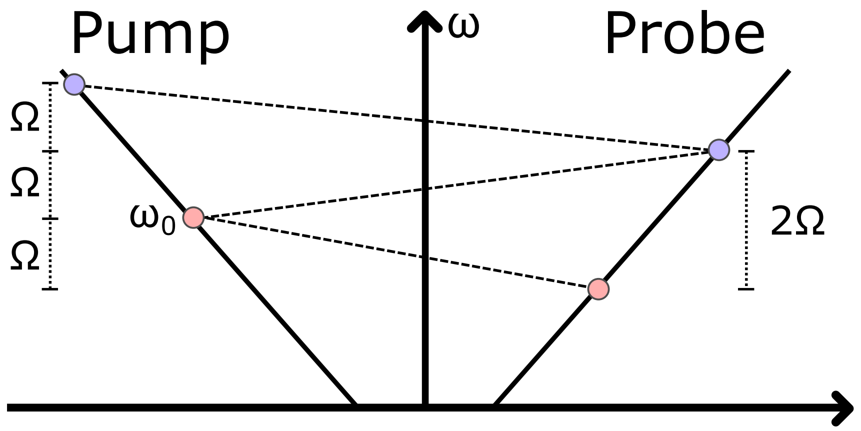

Fig. S8: Dispersion diagram with two optical pump-probe pairs, which a separated by twice the Brillouin frequency Ω. As a result, two Brillouin process are induced. Firstly, the pump with frequency ω_0 interacts with its corresponding probe field at frequency $\omega_0 - \Omega$. Secondly, the pump field also interacts with the probe field at $\omega_0 + \Omega$.

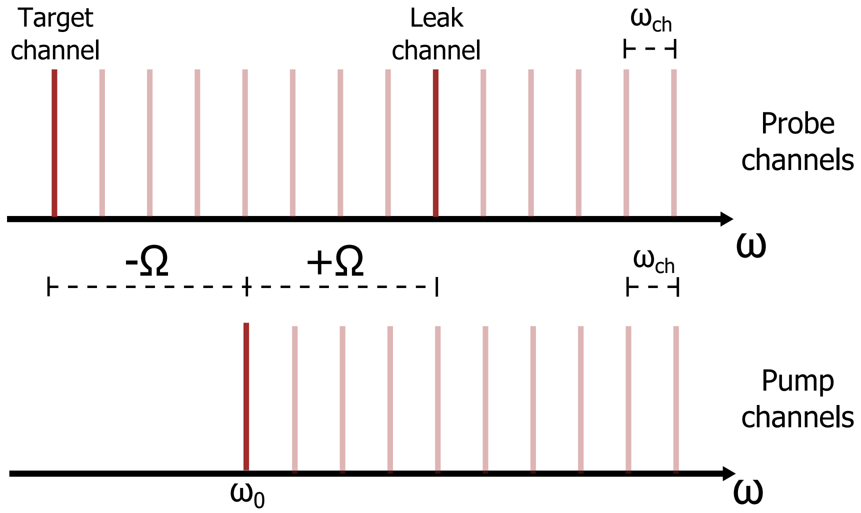

Fig. S9: Wavelength division multiplexed operation of the nonlinear activation function without frequency batching. The different frequency channels are separated by ω_{ch}. For the shown configuration, a pump wave would interact with two input probe channels because each is separated by the Brillouin frequency Ω to the pump. The Brillouin process would amplify the target channels whereas the twin channel would be attenuated.

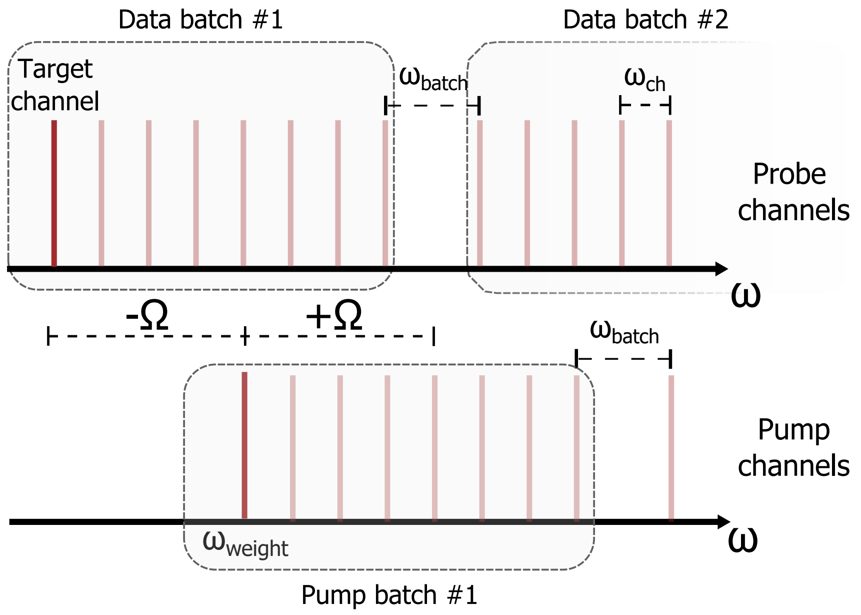

Fig. S10: Wavelength division multiplexed operation of the nonlinear activation function with frequency batching. The different frequency channels are separated by ω_{ch}. As the batches are separated by a batching frequency $\omega_{\mathrm{batch}} \neq l \cdot \omega_{\mathrm{ch}},\ l \in \mathbb{N}$, an optical pump interacts only with the target probe channel.
